# Effect of systemic vascular resistance on the agreement between stroke volume by non-invasive pulse wave analysis and Doppler ultrasound in healthy volunteers

**DOI:** 10.1371/journal.pone.0302159

**Published:** 2024-05-07

**Authors:** Sole Lindvåg Lie, Jonny Hisdal, Marius Rehn, Lars Øivind Høiseth

**Affiliations:** 1 Norwegian Air Ambulance Foundation, Department of Research and Development, Oslo, Norway; 2 Institute of Clinical Medicine, University of Oslo, Oslo, Norway; 3 Section of Vascular Investigations, Oslo University Hospital, Oslo, Norway; 4 Air Ambulance Department, Division of Prehospital Services, Oslo University Hospital, Oslo, Norway; 5 Department of Anesthesia and Intensive Care Medicine, Division of Emergencies and Critical Care, Oslo University Hospital, Oslo, Norway; Hiroshima University: Hiroshima Daigaku, JAPAN

## Abstract

**Background:**

Stroke volume can be estimated beat-to-beat and non-invasively by pulse wave analysis (PWA). However, its reliability has been questioned during marked alterations in systemic vascular resistance (SVR). We studied the effect of SVR on the agreement between stroke volume by PWA and Doppler ultrasound during reductions in stroke volume in healthy volunteers.

**Methods:**

In a previous study we simultaneously measured stroke volume by PWA (SV_PWA_) and suprasternal Doppler ultrasound (SV_US_). We exposed 16 healthy volunteers to lower body negative pressure (LBNP) to reduce stroke volume in combination with isometric hand grip to elevate SVR. LBNP was increased by 20 mmHg every 6 minutes from 0 to 80 mmHg, or until hemodynamic decompensation. The agreement between SV_PWA_ and SV_US_ was examined using Bland-Altman analysis with mixed regression. Within-subject limits of agreement (LOA) was calculated from the residual standard deviation. SVR_US_ was calculated from SV_US_. We allowed for a sloped bias line by introducing the mean of the methods and SVR_US_ as explanatory variables to examine whether the agreement was dependent on the magnitude of stroke volume and SVR_US_.

**Results:**

Bias ± limits of agreement (LOA) was 27.0 ± 30.1 mL. The within-subject LOA was ±11.1 mL. The within-subject percentage error was 14.6%. The difference between methods decreased with higher means of the methods (-0.15 mL/mL, confidence interval (CI): -0.19 to -0.11, P<0.001). The difference between methods increased with higher SVR_US_ (0.60 mL/mmHg × min × L^-1^, 95% CI: 0.48 to 0.72, P<0.001).

**Conclusion:**

PWA overestimated stroke volume compared to Doppler ultrasound during reductions in stroke volume and elevated SVR in healthy volunteers. The agreement between SV_PWA_ and SV_US_ decreased during increases in SVR. This is relevant in settings where a high level of reliability is required.

## Introduction

Reliable measurements of stroke volume are essential for understanding cardiovascular dynamics and assessing hemodynamic changes in both clinical and research settings. Stroke volume estimated by pulse wave analysis (PWA) using the volume-clamp method is non-invasive, user-independent and provides beat-to-beat values for real-time assessment [1]. However, studies have raised concerns about PWA’s reliability during marked alterations in systemic vascular resistance (SVR) [1, 2]. Since the arterial pressure waveform is given by the interplay of stroke volume, SVR and vascular compliance, we need to understand how SVR influences stroke volume estimated by PWA [3].

Lower body negative pressure (LBNP) and isometric handgrip (IHG) are two well-known methods that induce changes in SVR in healthy volunteers [4], potentially affecting the reliability of PWA estimated stroke volume. While LBNP primarily reduces cardiac filling and stroke volume [5], and IHG increases arterial blood pressure, both increase SVR [4]. Previous work has reported conflicting results on whether PWA underestimates stroke volume reductions during LBNP [6–8]. Also, two of the previous studies only applied mild-to moderate LBNP and thereby only minor alterations in SVR limiting their external validity. One study involving IHG concluded that stroke volume estimated by PWA was different from stroke volume measured by Doppler ultrasound although SVR did not increase with IHG [7]. Therefore, the effect of SVR on the reliability of PWA-estimated stroke volume remains uncertain. To our knowledge, no prior studies have examined the reliability of PWA-estimated stroke volume during reductions in stroke volume induced by LBNP in combination with IHG to maximize the increase in SVR. In a recent study we exposed healthy volunteers to LBNP and IHG, and stroke volume was simultaneously estimated by PWA and measured by Doppler ultrasound [9].

The aim of the present analysis was to study the effect of SVR on the agreement between stroke volume by PWA and suprasternal Doppler ultrasound during reductions in stroke volume with LBNP. We hypothesized that the agreement between stroke volume by PWA and suprasternal Doppler ultrasound would depend on SVR.

## Methods

### Subjects

The study was approved by the regional ethics committee (REC South East A, ref. 2017/136). Healthy volunteers > 18 years of age were recruited from November 1, 2017, to June 20, 2018. They provided written informed consent. The exclusion criteria were pregnancy, conditions limiting physical performance or requiring regular medication (except contraceptives) or history of syncope (except presumed vasovagal syncope). Subjects refrained from heavy physical activity and caffeine on the test day. Test sessions were conducted between 8 a.m. and 4 p.m.

### Experimental design

**Fig 1:**
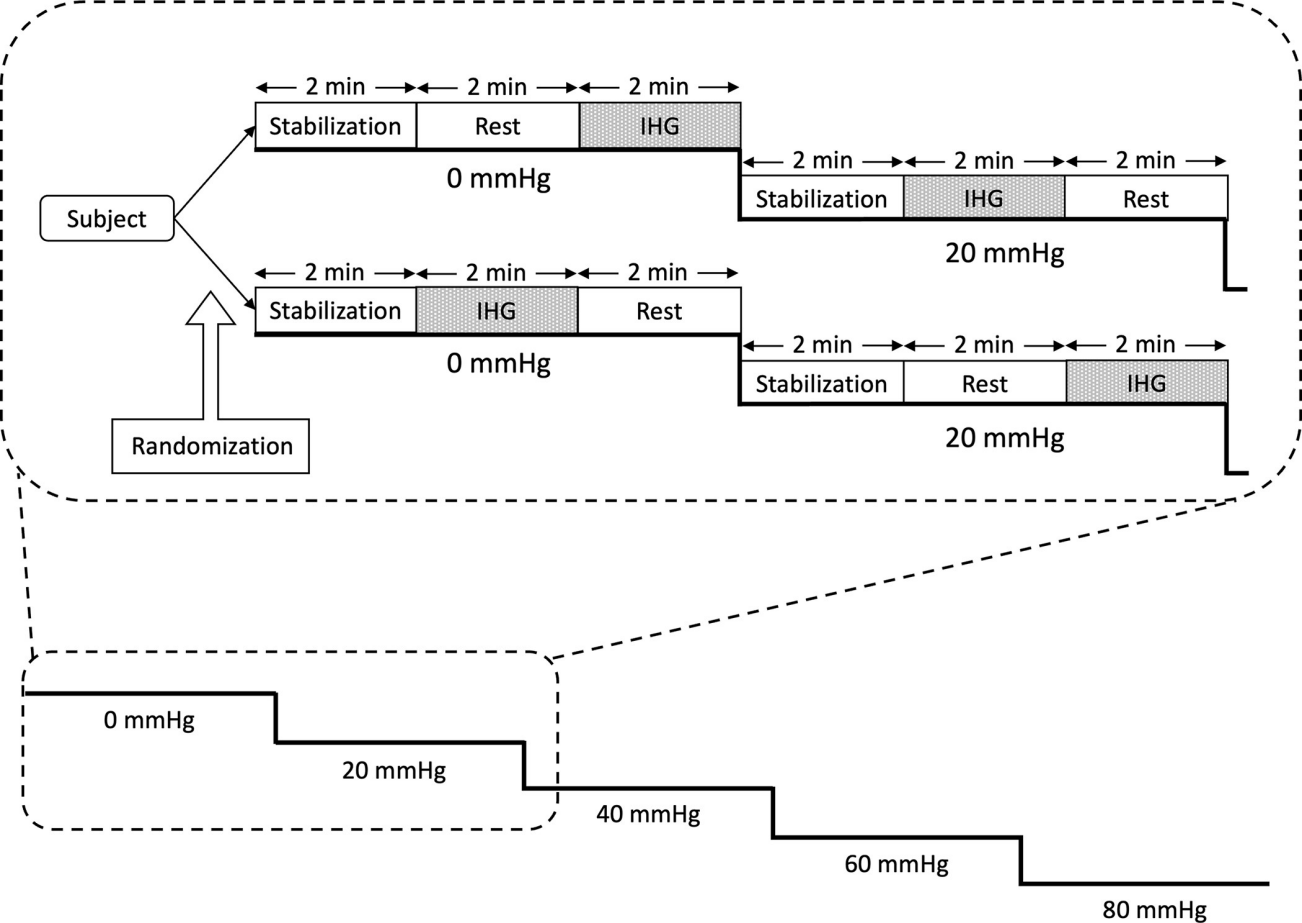
The experimental design. Subjects were randomized to begin LBNP 0 with isometric handgrip (IHG) or rest (no IHG) after the stabilization period. Lower body negative pressure (LBNP) was increased in levels of 20 mmHg from 0 to 80 mmHg (or until hemodynamic decompensation) with alternating IHG on every LBNP level.

Study design and presentation of data for a different research question has previously been published [9]. On the test day, subjects were accustomed with the setup and acclimatized for 20–30 minutes while in the supine position inside the LBNP chamber. Subjects underwent LBNP in 6-minute levels of 20 mmHg increments from 0 to 80 mmHg, or until signs or symptoms of presyncope such as a sudden drop in blood pressure or heart rate, light headedness, or nausea. LBNP could also be terminated on subject’s request for reasons other than those mentioned above. On every LBNP level the subject performed IHG. Each 6-minute LBNP level comprised three 2-minute periods, where the first was considered a stabilization period and the latter two either exposure to IHG or rest (no IHG) in an alternating fashion (Fig 1). The subjects were block-randomized to start with IHG or rest on the first LBNP level with block sizes 2 or 4 using the “blockrand” package in R [10]. Only completed LBNP levels were entered in the statistical analyses (i.e. LBNP levels with signs or symptoms of presyncope were excluded from analyses).

### Interventions

**Fig 2:**
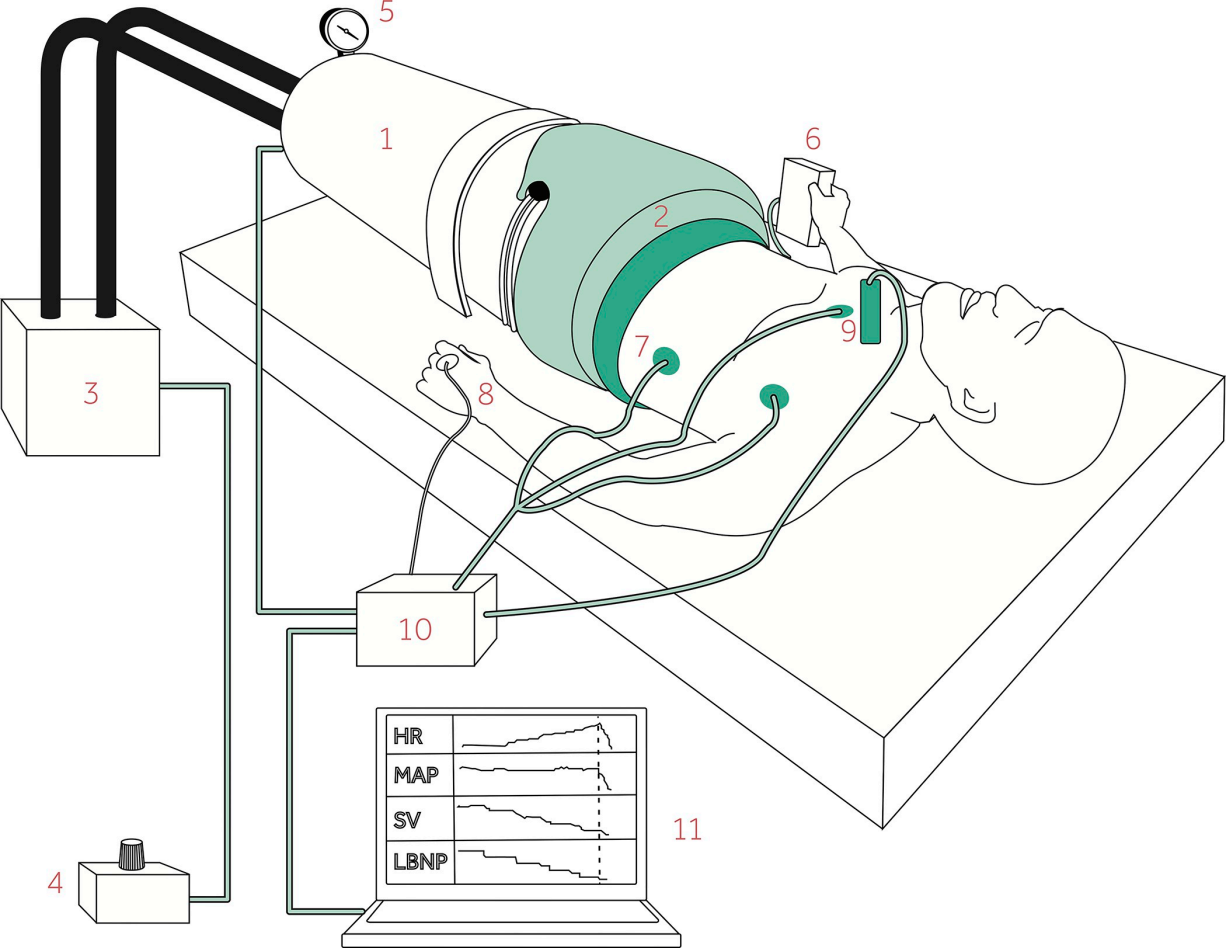
Lower body negative pressure (LBNP) model. The subject was placed inside 1) the LBNP chamber, which was 2) sealed just above the iliac crest and connected to 3) a vacuum pump controlled by 4) a pressure control unit. The applied negative pressure was displayed on 5) a pressure monitor. The subject squeezed 6) a hand grip device with the right hand. 7) Heart rate (HR), 8) mean arterial pressure (MAP) and 9) stroke volume (SV) were recorded on 10) a Bio Amp/PowerLab and 11) sampled on a laptop continuously. Reprinted from [12] under a CC BY license, with permission from the author.

Subjects were placed in the supine position inside the LBNP chamber [11] which was sealed at the level of the iliac crest (Fig 2). The subjects performed IHG by gripping their right hand on a force sensitive handle with visual feedback of the applied force. Maximum voluntary contraction for each subject was calculated as the average of three attempts measured before beginning the LBNP protocol. The subjects were instructed to keep 40% of this force for the 2-minute IHG periods, only engaging right forearm muscles and otherwise stay relaxed.

### Measurements

Heart rate (HR) was obtained from a three-lead ECG using Bio Amp/ PowerLab (ADInstruments, Bella Vista, Australia). Mean arterial pressure (MAP) was calculated as the time weighted integral of the arterial blood pressure waveform, measured with the volume-clamp method on the third finger of the left hand (Nexfin; BMEYE, Amsterdam, The Netherlands).

#### Stroke volume estimated by PWA

Stroke volume was estimated by PWA (SV_PWA_) of the arterial pressure waveform measured by the Nexfin device (Nexfin; BMEYE, Amsterdam, The Netherlands). The algorithm primarily analyzes the systolic part of the arterial pressure waveform and incorporates a three-element Windkessel model to determine aortic impedance and estimate stroke volume [13]. The device is internally calibrated by incorporating age, sex, height, and weight in the algorithm.

#### Stroke volume measured by suprasternal Doppler ultrasound

Stroke volume (SV_US_) was calculated from the aortic blood velocity measured by pulsed Doppler ultrasound with a 2 MHz probe in the suprasternal notch (SD-50; Vingmed Ultrasound, Horten, Norway). The ultrasound probe was directed towards the aortic root and the sample volume depth set to obtain the highest possible peak velocity and held constant throughout the test session. The same trained operator performed all the Doppler ultrasound measurements. The validity of this method has been described previously [14]. The left ventricular outflow tract (LVOT) area was obtained from an echocardiographic examination measuring the diameter from the inner edge to inner edge in the parasternal long axis and assuming a circular LVOT area. The velocity-time integrals for every heartbeat were multiplied by LVOT area to calculate stroke volume.

#### Cardiac output

Cardiac output_US_ was calculated as the product of SV_US_ and heart rate.

#### Systemic vascular resistance

Systemic vascular resistance (SVR_US_) was calculated as MAP divided by cardiac output_US_.

### Data processing

All signals were sampled simultaneously in Lab Chart 8.1.9 (ADInstruments, Bella Vista, Australia) at 1000 Hz. Beat-to-beat values were defined by the R-R-interval from the ECG and exported as textfiles to R 4.1.0 (R Foundation for Statistical Computing, Vienna, Austria)/ RStudio 1.4.1717 (RStudio, Boston, MA, USA) for further processing. Mean values for every 30 seconds were calculated for the dataset used in the analyses, trimming the 5% highest and lowest values to remove noise objectively and reproducibly. Because the release of IHG induced extremely rapid hemodynamic changes, we removed 30 seconds of data from the time of IHG release on every LBNP level.

### Statistics

We examined the agreement between SV_US_ and SV_PWA_ using Bland-Altman analysis with mixed linear regression to account for repeated measurements within subjects [15]. The total variability in the model was used to calculate limits of agreement (LOA), while the within-subject variability (residual standard deviation) was used to calculate within-subject LOA. Within-subject percentage error was calculated as within-subject LOA divided by the mean of the averaged paired measurements.

Next, we allowed for a sloped bias line by regressing the difference between methods (SV_PWA_—SV_US_) on the mean of methods ([SV_PWA_ + SV_US_]/2). To examine if this effect was associated with changes in SVR_US_, we introduced SVR_US_ as an explanatory variable in the regression model, after checking for statistical significance in a bivariable model. As an exploratory analysis to investigate a potential influence of sex on the estimated effect of SVR_US_ on the difference between methods, we introduced sex as a categorical explanatory variable in a multivariable regression model with SVR_US_ and their interaction effect.

SVR, MAP and cardiac output are mathematically and physiologically coupled. To account for this dependency, we regressed the difference between methods on time throughout the IHG periods. We used time throughout the IHG periods as a proxy for SVR_US_, since SVR_US_ increases during IHG. Time was entered as a linear explanatory variable, and LBNP level clustered within subjects was entered as a random effect.

Regression assumptions were checked using QQ-plots, histograms, and plots of residuals versus predicted values. Precisions in SV_US_ and SV_PWA_ were calculated as 1.96 × the residual standard deviation (SD) using mixed regression with subjects as a random effect on data from LBNP 0 without IHG. All statistical analyses were performed in the software R (R 4.1.0, R Foundation for Statistical Computing, Vienna, Austria)/ RStudio 1.4.1717 (RStudio, Boston, MA, USA). Data are presented as mean ± SD unless otherwise stated. P-values <0.05 were considered statistically significant. Regression outputs can be found in the S1 Appendix.

## Results

Sixteen subjects (nine females) with age 24 ± 3 years, weight 71 ± 14 kg, height 177 ± 11 cm and body mass index 23 ± 3 kg/m^2^ participated in this study. All subjects completed LBNP 20, 15 subjects (eight females) completed LBNP 40, 11 subjects (five females) completed LBNP 60 and two subjects (one female) completed LBNP 80.

**Fig 3:**
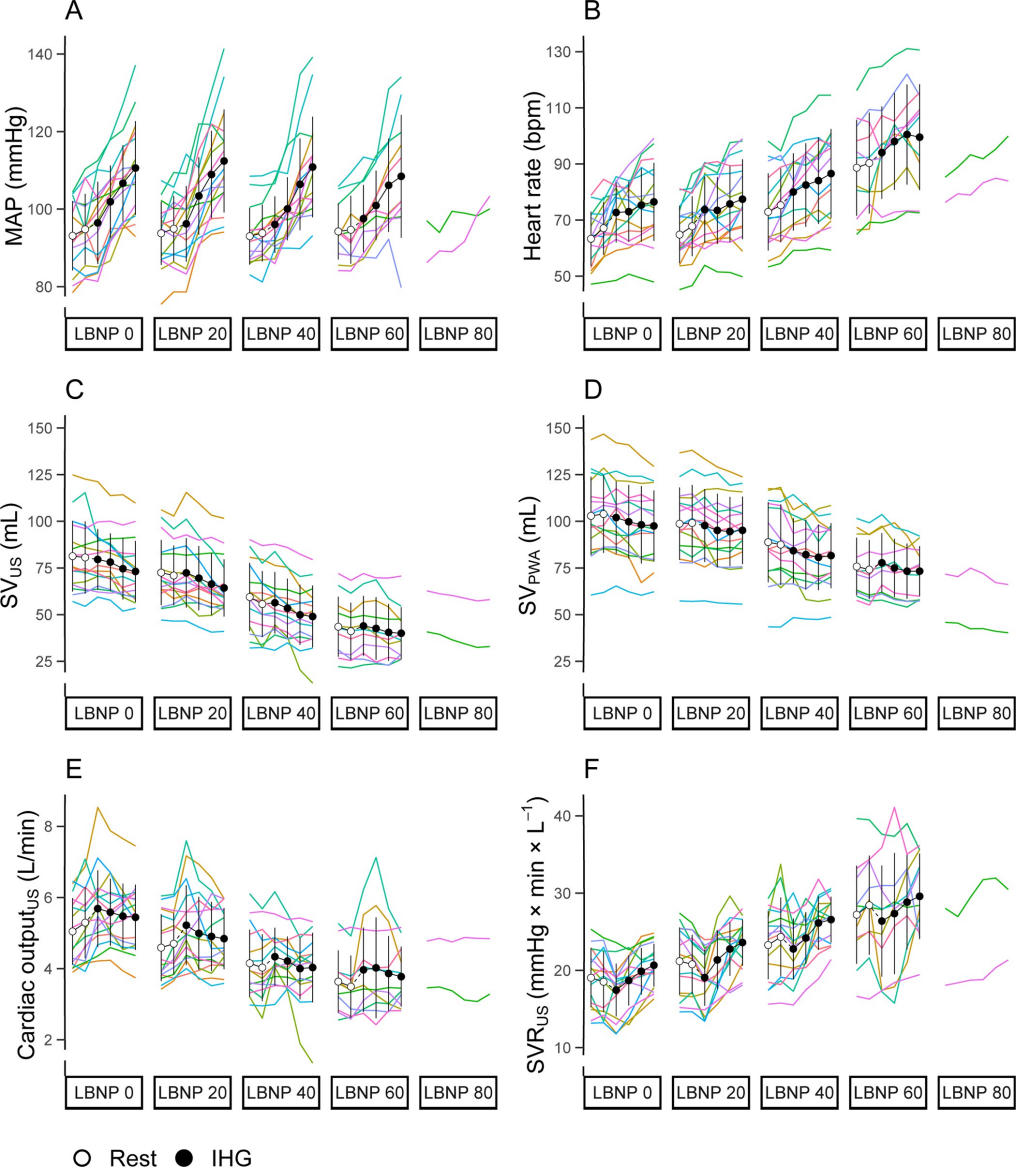
The hemodynamic response to lower body negative pressure (LBNP) and isometric handgrip (IHG). A) mean arterial pressure (MAP), B) heart rate, C) stroke volume measured by Doppler ultrasound (SV_US_), D) stroke volume estimated by pulse wave analysis (SV_PWA_), E) cardiac output from SV_US_ and F) systemic vascular resistance (SVR_US_). Circles and whiskers are mean ± standard deviation. Open circles represent rest and black circles represent IHG. Colored lines represent individual subjects.

The hemodynamic response to LBNP and IHG is presented in Fig 3, which shows the intended reduction in stroke volume with LBNP (SV_US_ and SV_PWA,_ Fig 3, panels C and D) and increase in MAP with IHG (Fig 3, panel A). SVR_US_ increased with LBNP and IHG (Fig 3, panel F). The precision in SV_US_ was ± 3.7 mL, and the precision in SV_PWA_ was ± 3.7 mL.

### Agreement between SV_PWA_ and SV_US_

**Fig 4:**
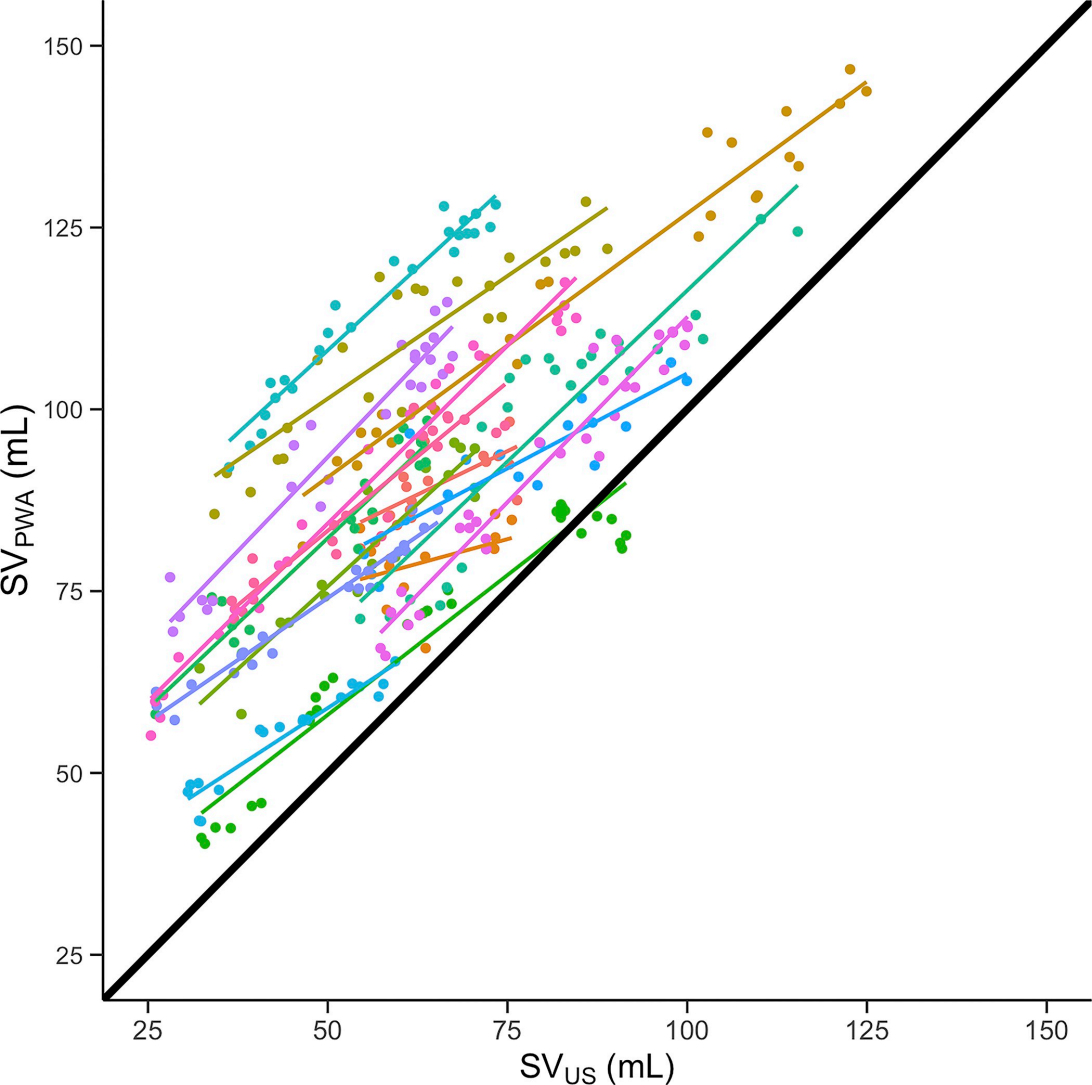
Scatterplot of stroke volume estimated by pulse wave analysis (SV_PWA_) against stroke volume measured by Doppler ultrasound (SV_US_). Colors represent subjects with individual simple linear regression lines. The black line shows the line of identity.

**Fig 5:**
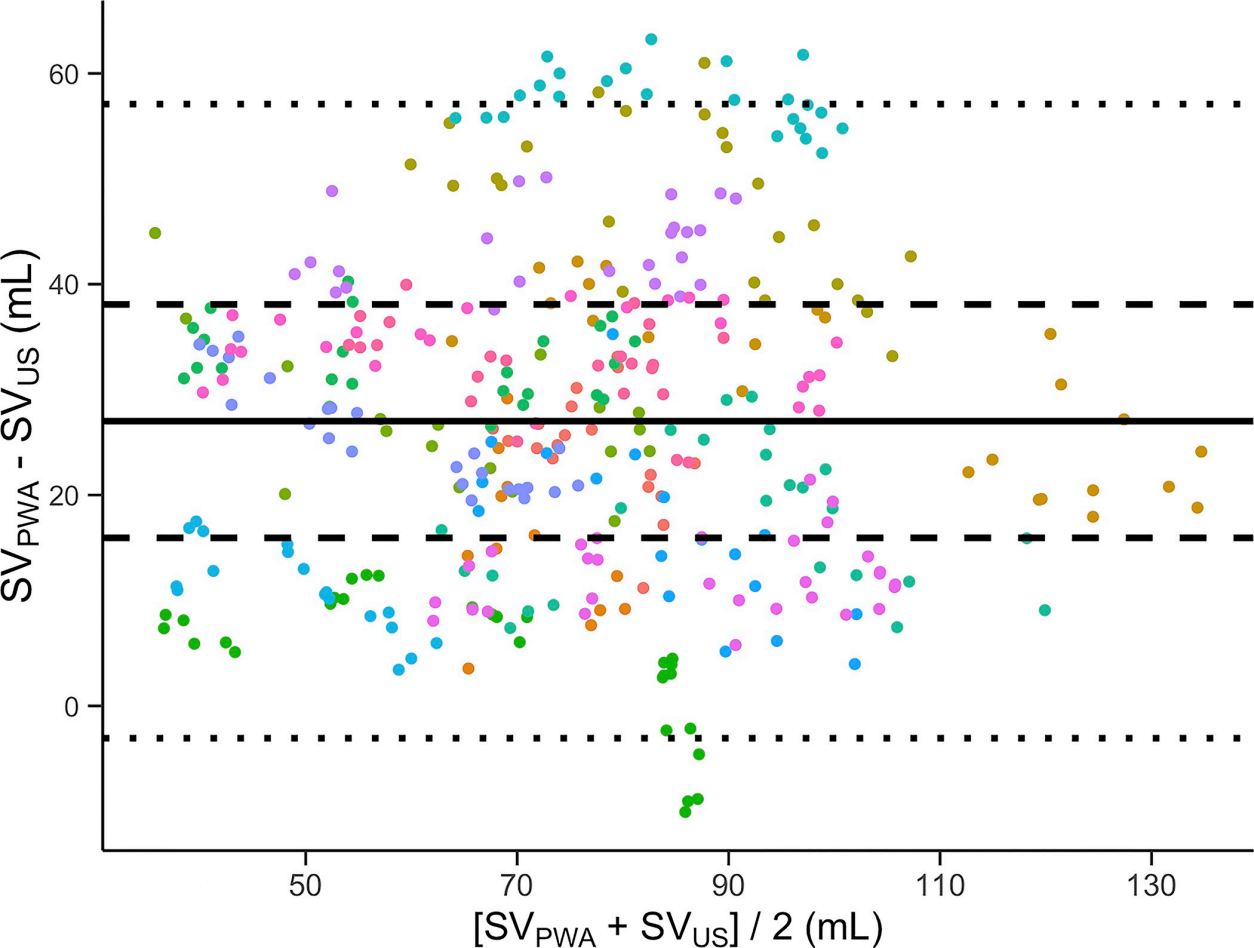
Bland-Altman plot with differences between methods (SV_PWA_—SV_US_) plotted against the mean of the methods ([SV_PWA_ + SV_US_]/2). Solid line is the bias, dotted lines are limits of agreement (LOA) from the total variance and dashed lines are the within-subject LOA. Colors represent subjects. SV_PWA_; stroke volume estimated by pulse wave analysis. SV_US_; stroke volume measured by Doppler ultrasound.

Fig 4 is a scatterplot of SV_PWA_ against SV_US_. In the Bland-Altman analysis, bias ± LOA was 27.0 ± 30.1 mL. The within-subject LOA was ± 11.1 mL (Fig 5). The within-subject percentage error was 14.6%.

### The effect of mean of the methods on agreement between SV_PWA_ and SV_US_

**Fig 6:**
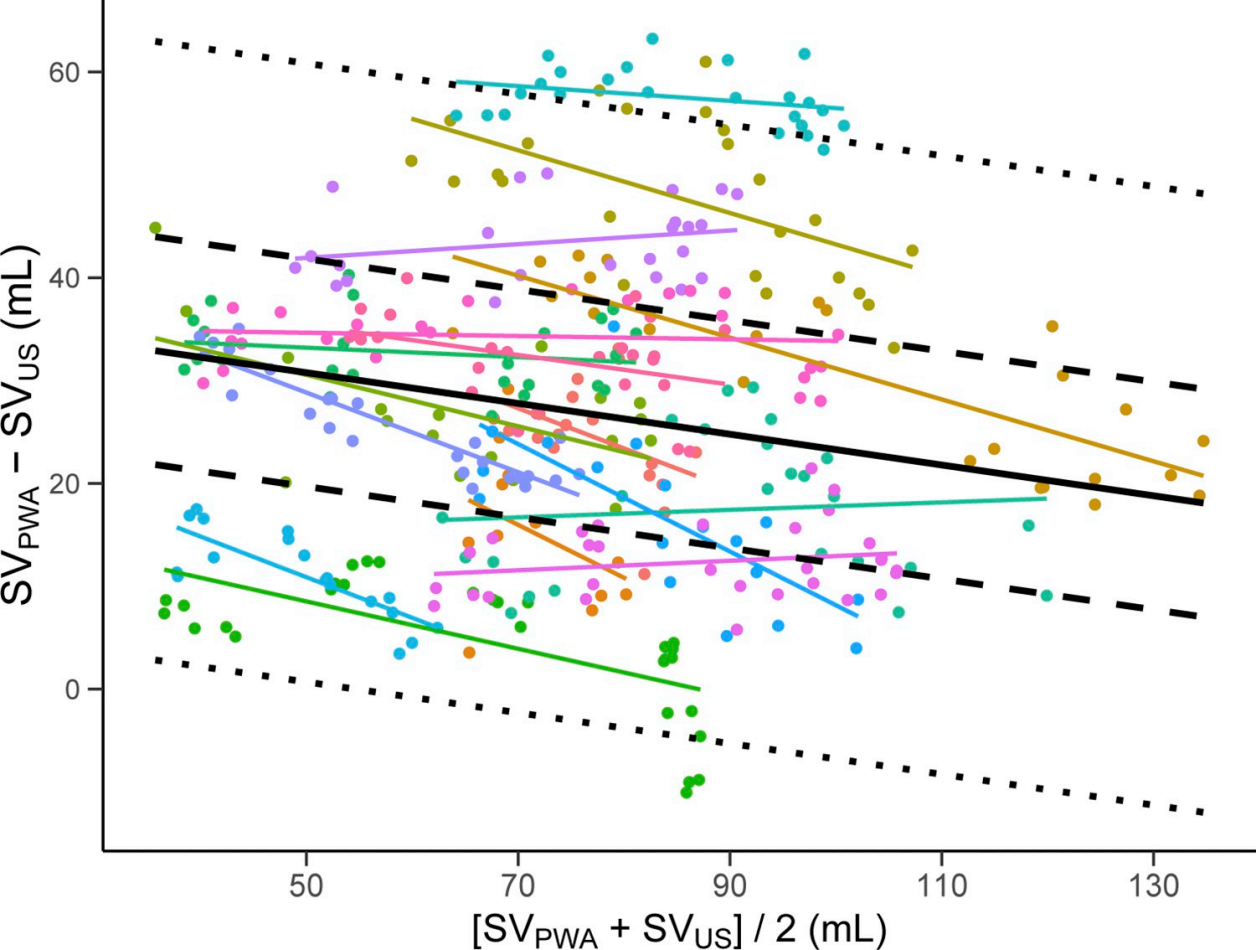
Bland-Altman plot with differences between methods (SV_PWA_—SV_US_) plotted against the mean of the methods ([SV_PWA_ + SV_US_]/2). Solid line is the bias which in this model is a function of the y-axis intercept and the mean of the methods. Dotted lines are limits of agreement (LOA) from the total variance and dashed lines are the within-subject LOA. Colors represent subjects with individual simple linear regression lines. SV_PWA_; stroke volume estimated by pulse wave analysis. SV_US_; stroke volume measured by Doppler ultrasound.

When allowing for a sloped regression line, the difference between methods decreased with higher means of the methods (-0.15 mL/mL, CI: -0.19 to -0.11, P < 0.001, Fig 6). LOA was ± 30.9 mL and within-subject LOA was ± 10.2 mL. The within-subject percentage error was 13.4%.

### The effect of SVR_US_ on the agreement between SV_PWA_ and SV_US_

**Fig 7:**
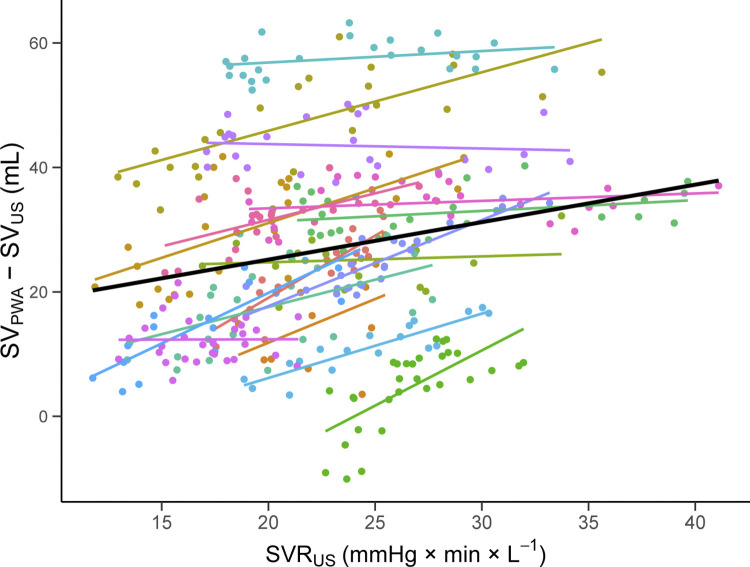
Scatterplot of the difference between methods (SV_PWA_—SV_US_) against systemic vascular resistance (SVR_US_). Solid line is the regression estimate for the effect of SVR_US_ on the difference between methods. Colors represent subjects with individual simple linear regression lines. SV_PWA_; stroke volume estimated by pulse wave analysis. SV_US_; stroke volume measured by Doppler ultrasound.

The difference between methods increased with higher SVR_US_ (0.60 mL/mmHg × min × L^-1^, 95% CI: 0.48 to 0.72, P <0.001, Fig 7). The explorative analysis revealed a greater influence of SVR_US_ in males compared to females (0.86 vs. 0.40 mL/mmHg × min × L^-1^, S1 Appendix).

### The effect of time during IHG periods on the agreement between SV_US_ and SV_PWA_

The difference between methods increased with time during IHG periods (0.58 mL/30 s, CI: 0.27 to 0.90, <0.001).

## Discussion

This experimental study in healthy volunteers demonstrated a statistically significant but small effect of SVR on the agreement between SV_PWA_ and SV_US_ during reductions in stroke volume and increases in SVR_US_ with LBNP and IHG. The difference between methods was lower when the means of the methods were higher, an effect explained by SVR_US_ in the regression analysis. This finding was further supported by a statistically significant effect of time during the IHG periods on agreement, since SVR_US_ increased with IHG. Our findings suggest that PWA overestimated absolute stroke volume values, and slightly underestimated reductions in stroke volume, compared to suprasternal Doppler ultrasound when SVR increased. Consequently, the agreement between SV_PWA_ and SV_US_ decreased during increases in SVR.

There was a consistent increase in the difference between methods when SVR_US_ increased (Fig 7). The average increase in SVR_US_ at maximum vasoconstriction was 10 mmHg × min × L^-1^, corresponding to an increased difference between methods of 6 mL. Our findings cohere with a previous study [7], although the observed effect in the present study was small. Compared to thermodilution, one study reported reliable stroke volume estimations by PWA during 30 mmHg of LBNP [6]. We expect that the discrepancy in study findings may be explained by the combination of LBNP and IHG, and the greater magnitude of LBNP, leading to larger SVR elevations in the present study. It might also be partially explained by employing different statistical methods, as we analyzed repeated measurements within subjects using a mixed regression model with SVR_US_ as an explanatory variable.

We investigated whether sex influenced the effect of SVR_US_ on agreement. Prior work indicates that SVR elevation during IHG is lower in females compared to males [16]. Our exploratory analysis revealed a statistically significant effect of sex on the difference between the methods, corresponding to a greater effect of SVR on agreement between SV_PWA_ and SV_US_ in males. Consequently, SVR appeared to have a diminished influence on the reliability of PWA-estimated stroke volume in females in our data. While this analysis was based on a small sample of nine females and should be interpreted cautiously, physiological differences between sexes might elucidate on the diverging study findings on PWA-estimated stroke volume in face of cardiovascular stressors that alter SVR.

SV_PWA_ was consistently higher than SV_US_ (Fig 4), corresponding to a large bias of 27.0 mL. In other words, PWA overestimated stroke volume compared to Doppler ultrasound both at rest and during LBNP and IHG. However, these numbers refer to the absolute values obtained by the devices. In recent years, there has been a shift in emphasis towards evaluating the trending abilities of devices–specifically, their capacity to monitor relative changes within individuals [2]. In such cases, the bias is less relevant and within-subject LOA is more important. In the present study the within-subject LOA was ± 11.1 mL, meaning that SV_PWA_ was more reliable when considering the method’s ability to track changes within individuals compared to between-subjects absolute values. This is further emphasized by the relatively small within-subject percentage error of 13.4%. Despite a large bias, our observations therefore suggest that PWA might be able to track reductions in stroke volume reliably within subjects.

In our study, potential data coupling may arise from multiple sources [17]. MAP, stroke volume, and SVR are interconnected as represented by the equation: MAP = stroke volume × heart rate × SVR (disregarding central venous pressure [CVP]). Additionally, data coupling arises when certain variables are measured using the same method, as in the case of SV_US_ and SVR_US_, both of which were determined using suprasternal Doppler ultrasound. Furthermore, there is a physiological data coupling since the mentioned hemodynamic variables are not entirely independent from one another. As a result, basic statistical assumptions may be compromised when assessing the impact of SVR_US_ on the agreement between SV_PWA_ and SV_US_. To address this, we used a proxy variable for SVR_US_ in our regression analyses to ascertain the consistency of the effect of SVR_US_ on agreement. Given that SVR_US_ increased during IHG periods, we substituted SVR_US_ with time during these periods. The results were consistent, implying a genuine influence of SVR on agreement, and suggesting that data coupling did not invalidate the findings in the present study.

According to a recent meta-analysis, non-invasive PWA is not interchangeable with invasive techniques to obtain cardiac output in adult surgical and critically ill patients [2]. The authors of this meta-analysis did however acknowledge the limitation of not evaluating the trending capabilities of PWA. Consequently, while PWA might demonstrate a substantial bias and broad LOA, it could still possess satisfactory trending abilities, as reflected in the within-subject LOA in the present study.

The PWA algorithms used in the Nexfin has been incorporated into the ClearSight system (Edwards Lifesciences, Irvine, CA) for clinical application. A recent study reported a substantial influence of SVR on the reliability of the ClearSight during changes in vascular tone by infusion of phenylephrine [18]. The pressor response induced by IHG, leading to an elevation in SVR, exhibits some similarities to the effects of phenylephrine. Importantly, we only found a small effect of SVR on the discrepancy between SV_PWA_ and SV_US_. While these authors reported a substantial impact of SVR on PWA-estimated stroke volume, it is worth noting that their statistical methods differed from those employed in the present study, as we estimated the effect of SVR on agreement by entering SVR_US_ as an explanatory variable using mixed regression analysis. This may complicate a direct comparison of results.

### Methodological considerations

Although Bland-Altman analyses do not assume a reference method, we calculated SVR from stroke volume measured by suprasternal Doppler ultrasound. This method offers direct measurement of blood velocity in the ascending aorta and allows for beat-to-beat recordings. The method has been validated previously [14]. As mentioned above, data coupling would have been a greater concern if we used PWA to acquire both MAP and SV when calculating SVR. Consequently, we utilized SV_US_ to determine SVR_US_. We believe it is more likely that PWA overestimated stroke volume, rather than suprasternal Doppler ultrasound consistently underestimated stroke volume. When standardizing SV_US_ to body surface area (BSA), the mean BSA-indxed SV_US_ of 43.2 mL/m^2^ at rest before LBNP in the present study was comparable to 38.7 mL/m^2^ observed in a healthy population [19]. While under different conditions, previous studies have also reported higher values with SV_PWA_ compared to SV_US_ [7, 8, 20]. Nonetheless, this potential deviation does not influence the within-subject LOA in our Bland-Altman analysis, nor the slope of the regression line in the scatterplot of difference between methods against SVR_US_.

We did not measure CVP, and in our calculation of SVR_US_, we made the assumption of a constant CVP using the equation: MAP—CVP = cardiac output × SVR_US_. However, CVP drops by approximately 1 mmHg per 10 mmHg of LBNP [5]. This results in an underestimation of the increase in SVR_US_, which has the potential to attenuate the influence of SVR_US_ on the agreement in the regression models. However, we found a statistically significant effect of SVR_US_. Therefore, we believe the observed effect of SVR_US_ on the agreement between SV_PWA_ and SV_US_ remains credible despite the assumption of constant CVP.

## Conclusion

In healthy volunteers, during a combination of LBNP and IHG to reduce stroke volume and increase SVR, the agreement between stroke volume by PWA and suprasternal Doppler ultrasound decreased during increases in SVR. Consequently, the agreement depended on SVR. PWA overestimated stroke volume compared to Doppler ultrasound, illustrated by the large bias of 27.0 mL. SVR had a small estimated effect on the agreement with an increased discrepancy between methods of 6 mL at maximum observed vasoconstriction with combined LBNP and IHG. Nonetheless, the influence of SVR might be problematic in experimental studies with high demands for reliable measurements of stroke volume. Depending on the setting, marked alterations in SVR may necessitate a cautious approach when utilizing PWA to estimate stroke volume.

## Supporting information

S1 Appendix Regression outputs can be found in “S1 Appendix”. (DOCX)

## References

[1] Thomsen KK, Kouz K, Saugel B. Pulse wave analysis: basic concepts and clinical application in intensive care medicine. Current Opinion in Critical Care. 2023;29: 215. doi: 10.1097/MCC.0000000000001039 37078625

[2] Saugel B, Hoppe P, Nicklas JY, Kouz K, Körner A, Hempel JC, et al. Continuous noninvasive pulse wave analysis using finger cuff technologies for arterial blood pressure and cardiac output monitoring in perioperative and intensive care medicine: a systematic review and meta-analysis. Br J Anaesth. 2020;125: 25–37. doi: 10.1016/j.bja.2020.03.013 32475686

[3] Saugel B, Kouz K, Scheeren TWL, Greiwe G, Hoppe P, Romagnoli S, et al. Cardiac output estimation using pulse wave analysis-physiology, algorithms, and technologies: a narrative review. Br J Anaesth. 2021;126: 67–76. doi: 10.1016/j.bja.2020.09.049 33246581

[4] Stens NA, Hisdal J, Bakke EF, Kaur N, Sharma A, Stranden E, et al. Factors mediating the pressor response to isometric muscle contraction: An experimental study in healthy volunteers during lower body negative pressure. Cipriano G, editor. PLoS ONE. 2020;15: e0243627. doi: 10.1371/journal.pone.0243627 33296410 PMC7725372

[5] Goswami N, Blaber AP, Hinghofer-Szalkay H, Convertino VA. Lower Body Negative Pressure: Physiological Effects, Applications, and Implementation. Physiological reviews. 2018/12/13 ed. 2019;99: 807–851. doi: 10.1152/physrev.00006.2018 30540225

[6] Shibasaki M, Wilson TE, Bundgaard-Nielsen M, Seifert T, Secher NH, Crandall CG. Modelflow underestimates cardiac output in heat-stressed individuals. American Journal of Physiology-Regulatory, Integrative and Comparative Physiology. 2011;300: R486–R491. doi: 10.1152/ajpregu.00505.2010 21084673 PMC3043797

[7] Dyson KS, Shoemaker JK, Arbeille P, Hughson RL. Modelflow estimates of cardiac output compared with Doppler ultrasound during acute changes in vascular resistance in women. Exp Physiol. 2010;95: 561–568. doi: 10.1113/expphysiol.2009.050815 20080867

[8] Holme NLA, Rein EB, Elstad M. Cardiac stroke volume variability measured non-invasively by three methods for detection of central hypovolemia in healthy humans. Eur J Appl Physiol. 2016;116: 2187–2196. doi: 10.1007/s00421-016-3471-2 27614883

[9] Lie SL, Hisdal J, Høiseth LØ. Cerebral blood flow velocity during simultaneous changes in mean arterial pressure and cardiac output in healthy volunteers. Eur J Appl Physiol. 2021;121: 2207–2217. doi: 10.1007/s00421-021-04693-6 33890157 PMC8260418

[10] Snow G. blockrand: Randomization for block random clinical trials (R package version 1.3, 2013). 2020. Available: https://CRAN.R-project.org/package=blockrand

[11] Hisdal J, Toska K, Walløe L. Design of a chamber for lower body negative pressure with controlled onset rate. Aviat Space Environ Med. 2003;74: 874–878. 12924764

[12] Lindvåg Lie S, Hisdal J, Rehn M, Høiseth LØ. Effects of supplemental oxygen on systemic and cerebral hemodynamics in experimental hypovolemia: Protocol for a randomized, double blinded crossover study. PLoS One. 2022;17: e0270598. doi: 10.1371/journal.pone.0270598 35749486 PMC9231698

[13] Truijen J, van Lieshout JJ, Wesselink WA, Westerhof BE. Noninvasive continuous hemodynamic monitoring. J Clin Monit Comput. 2012;26: 267–278. doi: 10.1007/s10877-012-9375-8 22695821 PMC3391359

[14] Eriksen M, Walløe L. Improved method for cardiac output determination in man using ultrasound Doppler technique. Med Biol Eng Comput. 1990;28: 555–560. doi: 10.1007/BF02442607 2287179

[15] Parker RA, Weir CJ, Rubio N, Rabinovich R, Pinnock H, Hanley J, et al. Application of Mixed Effects Limits of Agreement in the Presence of Multiple Sources of Variability: Exemplar from the Comparison of Several Devices to Measure Respiratory Rate in COPD Patients. PLoS One. 2016;11: e0168321. doi: 10.1371/journal.pone.0168321 27973556 PMC5156413

[16] Samora M, Incognito AV, Vianna LC. Sex differences in blood pressure regulation during ischemic isometric exercise: the role of the β-adrenergic receptors. J Appl Physiol (1985). 2019;127: 408–414. doi: 10.1152/japplphysiol.00270.2019 31219771 PMC6732446

[17] Archie JP. Mathematic coupling of data: a common source of error. Ann Surg. 1981;193: 296–303. doi: 10.1097/00000658-198103000-00008 7212790 PMC1345065

[18] Mukai A, Suehiro K, Kimura A, Tanaka K, Yamada T, Mori T, et al. Effect of Systemic Vascular Resistance on the Reliability of Noninvasive Hemodynamic Monitoring in Cardiac Surgery. J Cardiothorac Vasc Anesth. 2021;35: 1782–1791. doi: 10.1053/j.jvca.2020.11.011 33279380

[19] Patel HN, Miyoshi T, Addetia K, Henry MP, Citro R, Daimon M, et al. Normal Values of Cardiac Output and Stroke Volume According to Measurement Technique, Age, Sex, and Ethnicity: Results of the World Alliance of Societies of Echocardiography Study. J Am Soc Echocardiogr. 2021;34: 1077–1085.e1. doi: 10.1016/j.echo.2021.05.012 34044105 PMC9149664

[20] van Campen C Linda MC, Verheugt FWA, Rowe PC, Visser FC. Comparison of the finger plethysmography derived stroke volumes by Nexfin CO Trek and suprasternal aortic Doppler derived stroke volume measurements in adults with myalgic encephalomyelitis/chronic fatigue syndrome and in healthy controls. Technology and Health Care. 2021;29: 629–642. doi: 10.3233/THC-202669 33998565

