# Supplementary material for: Effect of systemic vascular resistance on the agreement between stroke volume by non-invasive pulse wave analysis and Doppler ultrasound in healthy volunteers
Source: PLoS One. 2024 May 7;19(5):e0302159. doi: 10.1371/journal.pone.0302159 (PMC11075826; doi:10.1371/journal.pone.0302159)
Supplement: S1 Appendix — (DOCX) [file pone.0302159.s001.docx]

Supplementary Material for “Effect of systemic vascular resistance on the agreement between stroke volume by non-invasive pulse wave analysis and Doppler ultrasound in healthy volunteers”

2024-03-20

In this appendix we provide the regression outputs for the reported results in the published paper. We examined the agreement between SV_US_ and SV_PWA_ using Bland-Altman analysis with mixed linear regression to account for repeated measurements within subjects. Subjects were entered as a random intercept.

# Abbreviations

PWA – pulse wave analysis

SV_US_ – stroke volume measured by suprasternal Doppler ultrasound

SV_PWA_ – stroke volume estimated by PWA

LOA – limits of agreement

SVR_US_ – systemic vascular resistance, calculated as MAP / (SV_US_ * heart rate)

# Precision

## Precision in SV_US_

|  | **SV_US_** | | | |
| --- | --- | --- | --- | --- |
| *Predictors* | *Estimates* | *SE* | *95% CI* | *p-val* |
| (Intercept) | 81.31 | 6.53 | 67.84 to 94.77 | **<0.001** |
| **Random Effects** | | | | |
| σ^2^ | 3.51 | | | |
| τ_00_ _ID_rand_ | 339.81 | | | |
| N _ID_rand_ | 8 | | | |
| Observations | 32 | | | |
| Marginal R^2^ / Conditional R^2^ | 0.000 / NA | | | |
| AIC | 178.070 | | | |

Precision = 1.96 * sqrt(3.51) = 3.67 ≈ 3.7 mL

## Precision in SV_PWA_

|  | **SV_PWA_** | | | |
| --- | --- | --- | --- | --- |
| *Predictors* | *Estimates* | *SE* | *95% CI* | *p-val* |
| (Intercept) | 107.38 | 7.26 | 92.40 to 122.36 | **<0.001** |
| **Random Effects** | | | | |
| σ^2^ | 3.61 | | | |
| τ_00_ _ID_rand_ | 420.62 | | | |
| N _ID_rand_ | 8 | | | |
| Observations | 32 | | | |
| Marginal R^2^ / Conditional R^2^ | 0.000 / NA | | | |
| AIC | 180.245 | | | |

Precision = 1.96 * sqrt(3.61) = 3.72 ≈ 3.7 mL

# Summary of Bland-Altman analyses

| Model | Bias (mL) | Limits of agreement | | Within-subject percentage error (%) |
| --- | --- | --- | --- | --- |
|  |  | Total LOA (mL) | Within-subject LOA (mL) |  |
| SV_PWA_-SV_US_ ~ 1 | 27.0 | 30.1 | 11.1 | 14.6 |
| SV_PWA_-SV_US_ ~ ([SV_PWA_+SV_US_]/2 | * | 30.9 | 10.2 | 13.4 |

*The bias in this model is a function of the y-axis intercept and the mean of the methods.

# Agreement between SV_PWA_ and SV_US_

|  | **SV_PWA_ - SV_US_** | | | |
| --- | --- | --- | --- | --- |
| *Predictors* | *Estimates* | *SE* | *95% CI* | *p-val* |
| (Intercept) | 27.01 | 3.58 | 19.97 to 34.05 | **<0.001** |
| **Random Effects** | | | | |
| σ^2^ | 31.93 | | | |
| τ_00_ _ID_rand_ | 203.57 | | | |
| N _ID_rand_ | 16 | | | |
| Observations | 360 | | | |
| Marginal R^2^ / Conditional R^2^ | 0.000 / NA | | | |
| AIC | 2348.372 | | | |

Bias = 27 mL.

LOA = 1.96 * sqrt(31.93 + 203.57) = 30.07818 ≈ 30.1 mL

Within-subject LOA = 1.96 * sqrt(31.93) = 11.0753 ≈ 11.1 mL

Within-subject percentage error = Within-subject LOA / mean([SV_PWA_ + SV_US_]/2) * 100 = 14.6%

# The effect of mean of the methods on agreement between SV_PWA_ and SV_US_

|  | **SV_PWA_ - SV_US_** | | | |
| --- | --- | --- | --- | --- |
| *Predictors* | *Estimates* | *SE* | *95% CI* | *p-val* |
| (Intercept) | 38.25 | 3.99 | 30.41 to 46.09 | **<0.001** |
| [SV_PWA_ + SV_US_]/2 | -0.15 | 0.02 | -0.19 to -0.11 | **<0.001** |
| **Random Effects** | | | | |
| σ^2^ | 26.80 | | | |
| τ_00_ _ID_rand_ | 222.06 | | | |
| N _ID_rand_ | 16 | | | |
| Observations | 360 | | | |
| Marginal R^2^ / Conditional R^2^ | 0.248 / NA | | | |
| AIC | 2296.508 | | | |

LOA = 1.96 * sqrt(26.80 + 222.06) = 30.91958 ≈ 30.9 mL

Within-subject LOA = 1.96 * sqrt(26.80) = 10.14667 ≈ 10.2 mL

Within-subject percentage error = Within-subject LOA / mean([SV_PWA_ + SV_US_]/2) * 100 = 13.4%

# The effect of SVR_US_ on the agreement between SV_PWA_ and SV_US_

## Difference between devices regressed on SVR_US_

|  | **SV_PWA_ - SV_US_** | | | |
| --- | --- | --- | --- | --- |
| *Predictors* | *Estimates* | *SE* | *95% CI* | *p-val* |
| (Intercept) | 13.15 | 3.79 | 5.68 to 20.61 | **0.001** |
| SVR_us | 0.60 | 0.06 | 0.48 to 0.72 | **<0.001** |
| **Random Effects** | | | | |
| σ^2^ | 23.79 | | | |
| τ_00_ _ID_rand_ | 199.36 | | | |
| N _ID_rand_ | 16 | | | |
| Observations | 358 | | | |
| Marginal R^2^ / Conditional R^2^ | 0.313 / NA | | | |
| AIC | 2239.383 | | | |

## Difference between devices regressed on mean of the methods and SVR_US_ in a multivariable regression model

|  | **SV_PWA_ - SV_US_** | | | |
| --- | --- | --- | --- | --- |
| *Predictors* | *Estimates* | *SE* | *95% CI* | *p-val* |
| (Intercept) | 11.99 | 5.67 | 0.85 to 23.13 | **0.035** |
| [SV_PWA_ + SV_US_]/2 | 0.01 | 0.03 | -0.05 to 0.07 | 0.784 |
| SVR_US_ | 0.63 | 0.10 | 0.42 to 0.83 | **<0.001** |
| **Random Effects** | | | | |
| σ^2^ | 23.86 | | | |
| τ_00_ _ID_rand_ | 198.29 | | | |
| N _ID_rand_ | 16 | | | |
| Observations | 358 | | | |
| Marginal R^2^ / Conditional R^2^ | 0.312 / NA | | | |
| AIC | 2246.457 | | | |

Because [SV_PWA_ + SV_US_]/2 is not statistically significant we removed it from the model. The estimate was also minor.

## Difference between devices regressed on SVR_US_, sex and interaction

|  | **The effect of SVRUS on** | | | |
| --- | --- | --- | --- | --- |
| *Predictors* | *Estimates* | *SE* | *95% CI* | *p-val* |
| (Intercept) | 13.28 | 5.39 | 2.68 to 23.89 | **0.014** |
| SVR_us | 0.86 | 0.09 | 0.69 to 1.03 | **<0.001** |
| Sex [Female] | 0.98 | 7.30 | -14.67 to 16.64 | 0.895 |
| SVR_us * Sex [Female] | -0.47 | 0.12 | -0.70 to -0.24 | **<0.001** |
| **Random Effects** | | | | |
| σ^2^ | 22.83 | | | |
| τ_00_ _ID_rand_ | 181.24 | | | |
| N _ID_rand_ | 16 | | | |
| Observations | 358 | | | |
| Marginal R^2^ / Conditional R^2^ | 0.464 / NA | | | |
| AIC | 2222.706 | | | |

## Difference between devices regressed on SVR_US_ and interaction between SVR_US_ and sex

|  | **The effect of SVRUS on** | | | |
| --- | --- | --- | --- | --- |
| *Predictors* | *Estimates* | *SE* | *95% CI* | *p-val* |
| (Intercept) | 13.81 | 3.54 | 6.84 to 20.79 | **<0.001** |
| SVR_us | 0.86 | 0.08 | 0.69 to 1.02 | **<0.001** |
| SVR_us * Sex [Female] | -0.46 | 0.11 | -0.68 to -0.25 | **<0.001** |
| **Random Effects** | | | | |
| σ^2^ | 22.82 | | | |
| τ_00_ _ID_rand_ | 170.94 | | | |
| N _ID_rand_ | 16 | | | |
| Observations | 358 | | | |
| Marginal R^2^ / Conditional R^2^ | 0.498 / NA | | | |
| AIC | 2226.513 | | | |

# The effect of time during IHG periods on the agreement between SV_PWA_ and SV_US_

|  | **SV_PWA_ - SV_US_** | | | |
| --- | --- | --- | --- | --- |
| *Predictors* | *Estimates* | *SE* | *95% CI* | *p-val* |
| (Intercept) | 25.91 | 3.59 | 18.83 to 32.99 | **<0.001** |
| Time, 30 s bins | 0.58 | 0.16 | 0.27 to 0.90 | **<0.001** |
| **Random Effects** | | | | |
| σ^2^ | 15.14 | | | |
| τ_00_ _LBNP_level_ | 14.09 | | | |
| τ_00_ _ID_rand_ | 4.52 | | | |
| N _LBNP_level_ | 5 | | | |
| N _ID_rand_ | 16 | | | |
| Observations | 300 | | | |
| Marginal R^2^ / Conditional R^2^ | 0.043 / NA | | | |
| AIC | 1850.345 | | | |
